# Ethanol Administration in Mice Leads to Sex-Specific Changes in the Acetylation of α-Tubulin in the Cerebellum

**DOI:** 10.3390/brainsci15040326

**Published:** 2025-03-21

**Authors:** Abosede Elesinnla, Rehana Khatoon, Nicholas Kleinert, Junfang Wu, Jaylyn Waddell, Tibor Kristian

**Affiliations:** 1 Department of Anesthesiology and the Center for Shock, Trauma and Anesthesiology (S.T.A.R.), University of Maryland School of Medicine, 685 Baltimore Street, Baltimore, MD 21201, USA; aelesinnla@som.umaryland.edu (A.E.); rkhatoon@som.umaryland.edu (R.K.); junfang.wu@som.umaryland.edu (J.W.); 2 Veterans Affairs Maryland Health Center System, 10 North Greene Street, Baltimore, MD 21201, USA; nkleinert@som.umaryland.edu; 3 Department of Pediatrics, University of Maryland School of Medicine, 655 W. Baltimore St., Baltimore, MD 21201, USA; jwaddell@som.umaryland.edu

**Keywords:** ethanol, cerebellum, acetylation, acetyl-CoA, α-tubulin

## Abstract

Background: Acetylation of α-tubulin is an important post-translational modification that helps maintain microtubules’ stability and dynamics, including axonal transport, cell signaling, and overall neuronal integrity. This study investigates sex-based differences in alcohol-induced acetylation of α-tubulin in mouse cerebellum. Methods: Adult, 3-month-old male and female C57BL/6 mice were administered 20% ethanol intraperitoneally. The cerebellum was dissected at 30 min, 1 h, 2 h, and 4 h post-injection. Expression levels of cerebellar acetylation of α-tubulin and enzymes mediating acetylation/deacetylation were analyzed by Western blot. The downstream product of ethanol metabolism, acetyl-CoA, was quantified by HPLC. Results: In males, α-tubulin acetylation levels increased significantly as early as 30 min post-ethanol injection, whereas females exhibited increased acetylation at a later time point, after 1 h. These sex-specific changes coincided with alterations in acetyl-CoA levels that increased significantly at 15 min in males and 1 h in females following ethanol administration. Furthermore, the level of acetyltransferase that acetylates tubulin increased significantly at 30 min in males and 1 h in females. Notably, however, no significant changes were observed in the level of the tubulin deacetylating enzyme, HDAC6, in either sex. Conclusions: Our data demonstrate that these sex differences stem from variations in expression levels of tubulin acetyltransferase (αTAT1), and the rate of ethanol metabolism-related acetyl-CoA production between male and female animals.

## 1. Introduction

The acetylation of microtubules is a widely studied post-translational modification (PTM) of polymerized microtubules [1,2,3]. Recent studies have found that alcohol consumption affects various cellular processes in the brain, including the acetylation of α-tubulin [4,5]. The acetylation of α-tubulin ensures the stability of microtubules and their optimal performance within neurons. Microtubules play a vital role in various cellular processes, including cell division, the intracellular trafficking of organelles, cellular motility, mitochondrial dynamics, and maintaining the cell shape [6,7,8]. Therefore, understanding the impact of α-tubulin acetylation due to alcohol consumption is essential for uncovering its modulatory effects on neuronal functions and activity.

Recent studies have shown that alcohol consumption can affect the level of protein acetylation in mouse brain tissues leading to the modulation of specific signaling pathways [4,9]. The two enzymes that are involved in controlling the levels of α-tubulin acetylation are the histone deacetylase 6 (HDAC6), which deacetylates α-tubulin, and the α-tubulin N-acetyltransferase 1 (αTAT1), which transfers the acetyl group from acetyl-CoA to the lysine 40 residue of the α-tubulin [10]. The regulation of these enzymes affects the maintenance of proper cell physiology [11].

Sex differences in alcohol-induced α-tubulin acetylation in the mouse brain represents an important area of research since there are sex differences in ethanol metabolism [12]. The majority of studies that examined the effect of ethanol intake on brain tissue were performed using only males for the experiments [13,14,15]. The specific changes in the level of acetylated α-tubulin, the distinct expression levels of the key enzymes regulating α-tubulin acetylation (αTAT1 and HDAC6), and the variations in ethanol metabolism-dependent acetyl-CoA generation in male and female mouse brains are not fully understood. This study, therefore, aims to shed light on the differences in α-tubulin acetylation and its modulation by ethanol intake between male and female mice. Since we recently published data showing the sex-differences of ethanol on mitochondrial dynamics in Purkinje neurons, we focused this study on the cerebellum.

## 2. Materials and Methods

### 2.1. Animals

We used adult, 3-month-old, male and female C57BL/6 (strain: 000664) wild-type (WT) mice from Jackson Laboratories. The mice were assigned randomly to control (vehicle-injected) and ethanol-treated groups. Ethanol (20% in PBS, 2 g/kg) was administered intraperitoneally. We chose this dose to match the ethanol dose used in our previous study [12] and also in publications where effects of ethanol on brain tissue were reported [13,15,16]. At 30 min, 1, 2, and 4 h post-injection, the animals were decapitated and their cerebellum tissues dissected. A total of 84 mice were used for experiments. All animal experiments adhered to the highest standard outlined in the Guide for the Care and Use of Laboratory Animals of the National Institute of Health. Approval was obtained from the Animal Care and Use Committee of the University of Maryland, Baltimore. The animals were housed in temperature-controlled rooms at 23 °C and had unrestricted access to water and a maintenance diet. Furthermore, they were free of all viral, bacterial, and parasitic pathogens.

### 2.2. Brain Tissue Extraction and Protein Estimation

Following dissection of the cerebellum on ice, the tissue was homogenized in 2 mL of a lysis buffer composed of a RIPA buffer containing deacetylase inhibitors trichostatin A (TSA, 4 µL) and nicotinamide (Nam, 10 mM), and 20 µL of a protease and phosphatase inhibitor cocktail (PI). The homogenates were vortexed, briefly sonicated, and centrifuged at 10,000× *g* for 10 min at 4 °C. To conduct Western blot analysis, we estimated protein concentration in the supernatant using the Lowry assay.

### 2.3. Western Blot

For Western blots, 15–25 μg of the samples were treated with β-mercaptoethanol and Bio-Rad SDS-sample buffer. The mixture was heated to 95 °C and then separated into 4–15% BioRad Mini-PROTEAN TGX precast gel. The PVDF membranes were activated in 100% methanol for 1 min and then transferred into a dark box containing deionized water until needed. Using the advanced Trans-Blot Turbo system from BioRad (Hercules, CA, USA), the proteins were transferred onto the PVDF membrane (Millipore, Burlington, MA, USA). Afterward, the transblotted PVDF membrane was incubated overnight at 4 °C with the primary antibody of interest in a blocking buffer. The antibodies used include rabbit monoclonal HDAC6 (cell signaling, Danvers, MA, USA, #7612) at 1:3000, mouse β-actin (cell signaling, #3700) at 1:5000, rabbit αTAT1 (Invitrogen, Waltham, MA, USA, #PA5-114922) at 1:250, mouse α-tubulin (cell signaling, #3873), 1:20,000, AceCS1 (cell signaling, #3658) at 1:1000, and rabbit acetyl-α-tubulin (cell signaling, #5335) at 1:50,000.

The membranes were washed in PBS with 0.1% TWEEN-20 (PBS-T) and incubated for 1 h at room temperature with an appropriate secondary antibody tagged with an infrared probe (Li-cor Biosciences, Lincoln, NE, USA) at 1:10,000 dilution in PBS-T. We visualized the antibody immunoreactivity using the Azure Sapphire Biomolecular Imager (Azure Biosystems, Dublin, CA, USA) and quantified the various band intensities using AZURE Spot Pro software (Azure Biosystems, v1.4.583, Dublin, CA, USA). We conducted the Western blot analysis by normalizing the quantified bands of the protein of interest to β-actin or α-tubulin values.

### 2.4. Perchloric Acid Extraction

A separate group of animals were used to collect cerebellum samples for acetyl-CoA measurement using HPLC. After the cerebellum was dissected on ice, it was processed for acetyl-CoA measurement according to the protocol for the acetyl-CoA assay kit from Abcam (Waltham, MA, USA) (ab87546). Briefly, the tissue frozen in liquid nitrogen was weighed and homogenized in 2 µL of 1 N perchloric acid per milligram of tissue (average weight of mouse cerebellum was around 57 mg). The tubes were then placed on ice and incubated for 15 min while being periodically vortexed for 10 s every 2–3 min to maximize metabolite extraction. Afterward, the homogenates were centrifuged at 10,000× *g* for 10 min at 4 °C. The protein-free supernatant was then centrifuged again under the same conditions. The supernatant was neutralized with 3 M KHCO_3_ at 1 µL per 10 µL of supernatant to bring the pH to around 6–8. The neutralized supernatant was centrifuged at 10,000× *g* for 10 min, filtered, and frozen. The concentrations of acetyl-CoA in the samples were determined by HPLC and the values were normalized to the wet weight of the tissues.

### 2.5. High-Performance Liquid Chromatography (HPLC) Analysis

The HPLC system consists of the Agilent 1260 Infinity Quaternary LC, which uses a UV-VIS detector, 1260 MCT, 1260 multi-sampler (precooled to 4 °C before every run), and a 1260 Quaternary pump (Agilent, Santa Clara, CA, USA) with a C18 (3 × 150 mm, 120 Å) column (ThermoScientific, Waltham, MA, USA). The mobile phase comprises 100 mM monosodium phosphate and 75 mM sodium acetate. The mobile phase was filtered and degassed before analysis. All solvents used were HPLC-grade purity. We adjusted the mobile phase pH to 4.6 with phosphoric acid. Acetonitrile was added to the prepared mobile phase at a ratio of 94 (mobile phase) to 6 (acetonitrile) (*v*/*v*). We set the column temperature to 30 °C and the wavelength for UV detection to 259 nm. The flow rate was 0.5 mL/min, and the injection volume was 50 µL in each sample well using an isocratic method. The HPLC system used 30 µL of the injection volume. The standard stock solution of acetyl-CoA was obtained by serial dilution in 5% aqueous PCA ranging from 40 ng/µL to 1 ng/µL. Under these conditions, acetyl-CoA elute at 9.2 min, respectively—the run time was completed within 12 min. We ran blanks between sample runs to ensure purity. The concentrations of acetyl-CoA in each sample were calculated in nanomoles per milligram of wet tissue weight using the corresponding calibration curves.

### 2.6. Statistical Analysis

Statistical analysis of all data obtained in this study was performed using ANOVA in GraphPad Prism 10.2.2 software. Multiple comparisons were conducted using a post hoc Dunnett test. A *p*-value of less than 0.05 was considered statistically significant.

## 3. Results

### 3.1. Ethanol Induces Sex-Dependent Temporal Alterations in α-Tubulin Acetylation

**Figure 1:**
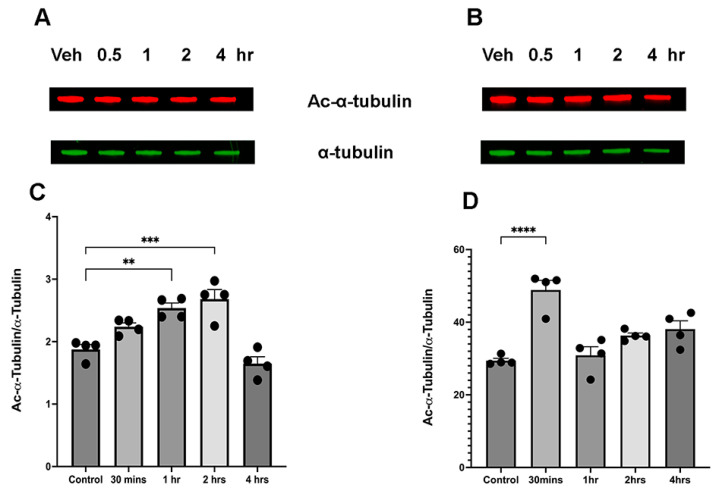
Temporal profile of ethanol-induced changes in a-tubulin acetylation. (**A**,**C**) Female cerebellum samples F(4,15) = 18.41, (**B**,**D**), male cerebellum samples F(4,15) = 15.74. ** *p* < 0.01, *** *p* < 0.001, **** *p* < 0.0001, one-way ANOVA for multiple groups (*n* = 4).

First, we compared the acute effect of ethanol on α-tubulin acetylation in male and female cerebella. The cerebellum was chosen because it is one of the most sensitive brain regions to ethanol toxicity [13,17,18]. As Figure 1 illustrates, the temporal profile of changes in α-tubulin acetylation after ethanol administration differs between male and female samples. In female samples, a significant increase in acetylation was observed at 1 and 2 h following ethanol administration (Figure 1A,C). In contrast, male samples showed a transient increase in α-tubulin acetylation as early as 30 min after ethanol injection (Figure 1B,D). Both male and female animals returned to normal acetylation levels at 1 h and 4 h after ethanol injection, respectively (Figure 1C,D).

### 3.2. α-Tubulin Acetylation Changes Coincide with an Ethanol-Induced Increase in Acetyl-CoA

**Figure 2:**
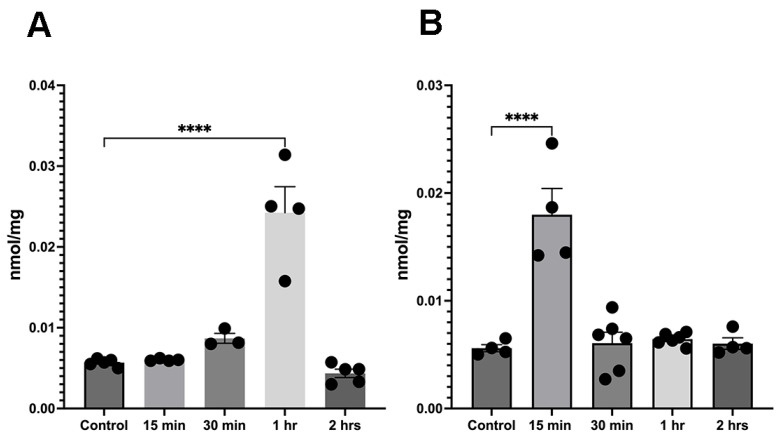
Ethanol administration leads to an increase in acetyl-CoA levels in cerebellar tissue. (**A**) The female cerebellum shows a significant increase 1 h after ethanol administration F(4,16) = 33.92. (**B**) In the male cerebellum, acetyl-CoA increased already at 15 min post-ethanol injection F(4,19) = 20.74. **** *p* < 0.0001, one-way ANOVA (*n* = 3–6).

Since the activity of acetyl transferases (KATs) is dependent on the availability of acetyl-CoA, we investigated the effects of ethanol administration on acetyl-CoA levels. In female mouse cerebellum, we detected an increase in acetyl-CoA at 1 h post-ethanol treatment (Figure 2A). However, in male mice, acetyl-CoA levels showed a significant increase 15 min post-ethanol treatment (Figure 2B). These changes were transient and were followed by the return of acetyl-CoA back to control levels.

### 3.3. Ethanol Induced Transient Changes in Acetyl-CoA Synthetase Levels Are Sex Dependent

**Figure 3:**
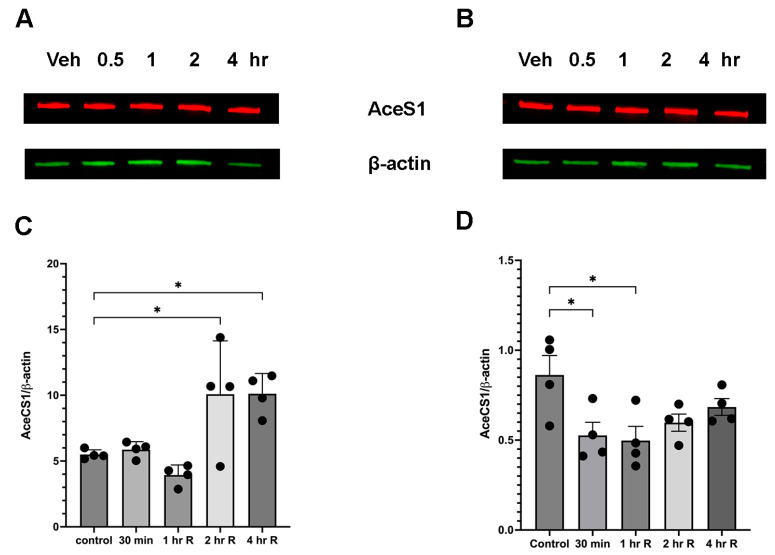
Ethanol administration leads to an increase in AceCS1 levels in female cerebellar tissue. (**A,C**) Female cerebellum shows a significant increase 2 h after ethanol administration F(4,15) = 8.5. (**B,D**) In male cerebellum there is a decrease in AceCS1 post-ethanol injection F(4,15) = 3.884 . * *p* < 0.05, one-way ANOVA (*n* = 4).

A downstream metabolite of ethanol degradation is acetate, and it serves as substrate for the generation of acetyl-CoA by acetyl-CoA synthetase (AceCS1). Therefore, we also examined the changes in the expression levels of this enzyme during the acute phase following ethanol administration (Figure 3). Interestingly, in female cerebellum there was an increase in AceCS1 levels at 2 and 4 h post-ethanol injection. However, in males the AceCS1 levels were reduced after ethanol treatment (Figure 3B,D).

### 3.4. Ethanol Administration Leads to Sex-Dependent Transient Increase in aTAT1 Expression

**Figure 4:**
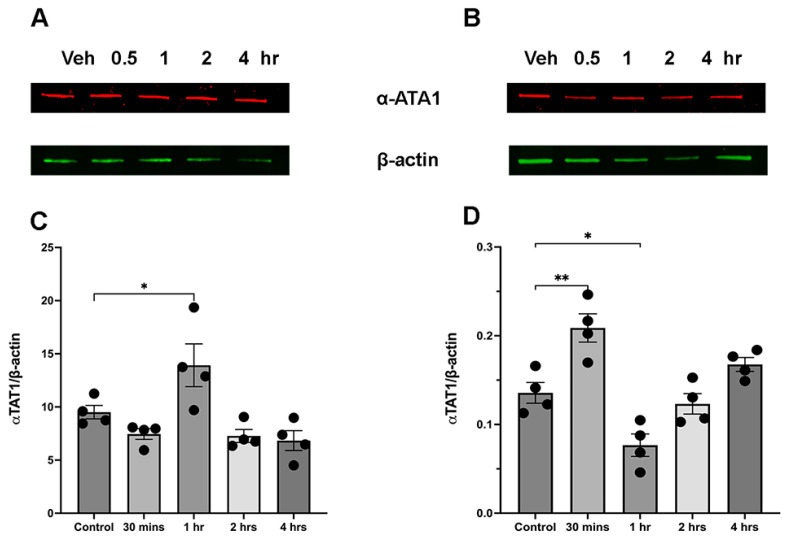
Sex-specific effects of ethanol administration on α-TAT1. (**A**,**C**) Female cerebella show a significant increase 1 h after ethanol administration F(4,15) = 7.27. (**B**,**D**) In male cerebellum α-TAT1 increased already at 30 min post-ethanol injection F(4,15) = 16.36. * *p* < 0.05, ** *p* < 0.01, one-way ANOVA for multiple groups (*n* = 4).

Given that the primary enzyme responsible for transferring the acetyl group from acetyl-CoA to the lysine residue of tubulin is αTAT1, we also determined the changes in αTAT1 expression levels following ethanol administration. In both male and female mice there was a transient increase in αTAT1. The αTAT1 enzyme showed a significant increase 30 min after ethanol administration in male cerebellum (Figure 4B,D) and at 1 h in female cerebellum (Figure 4A,C). In both males and females this was followed by the return of αTAT1 level to control levels (Figure 4C,D).

### 3.5. Ethanol Intake Does Not Affect the HDAC6 Expression Levels

**Figure 5:**
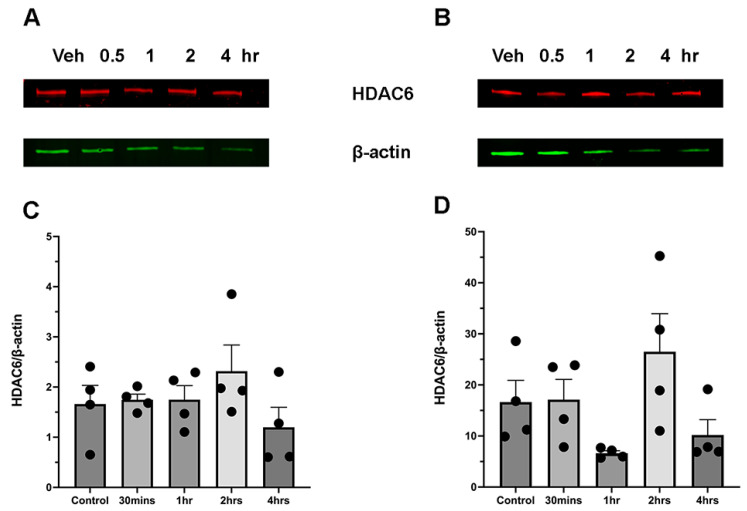
Ethanol administration does not affect HDAC6 expression levels. (**A**,**C**) Female cerebellum F(4,15) = 1.93. (**B**,**D**) Male cerebellum F(4,15) = 2.940. One-way ANOVA (*n* = 4).

Histone deacetylase 6 (HDAC6) is the major enzyme that deacetylates α-tubulin [19]. Figure 5 shows the impact of ethanol on the regulation of HDAC6 expression in the cerebellum of both male and female mice. There were no significant changes in the HDAC6 expression levels in the cerebellum of either male or female mice in the post-ethanol administration period (Figure 5C,D).

## 4. Discussion

Most of the ethanol that enters the body is metabolized by the liver due to the high expression of ethanol-degrading enzymes in this organ [20,21]. However, elevated serum ethanol levels and the high permeability of cellular membranes to ethanol result in its distribution to other organs, including the brain [22]. The metabolism of ethanol involves a series of biochemical reactions that generate acetate, which stimulates the formation of acetyl-CoA by acetyl-Co synthetase (AceCS). Elevated levels of acetyl-CoA serve as a substrate for KAT, leading to an increase in the acetylation of histone and non-histone proteins, including α-tubulin [23].

The presented data show a substantial rise in the level of acetylated α-tubulin in both the male and female cerebellum following ethanol administration. This finding corroborates previous studies that have also observed significant increases in acetylated α-tubulin throughout the entire brain of rats after ethanol consumption, as well as in post-mortem samples of the prefrontal cortex from alcoholic human brain tissue [9,24]. Furthermore, a related study reported ethanol induces α-tubulin acetylation in the rat liver [25,26].

Male mice exhibited a transient rise in α-tubulin acetylation at 30 min post-ethanol administration, which subsequently returned to the control level at the 1 h time point. In contrast, female mice displayed a more prolonged increase in acetylated α-tubulin from 1 h to 2 h after the ethanol treatment period. While several studies have reported an elevation in the level of acetylated α-tubulin following ethanol consumption [9,24], the observed differences in the temporal dynamics of acetylation between male and female mice in different brain regions were not determined. We recently reported that, 1 h after ethanol injection, the serum ethanol concentration in male mice was lower than in females [12]. This suggests that male mice metabolize ethanol more quickly and have shorter-lived elevated ethanol levels compared to females. Therefore, the varying temporal profile of protein acetylation can partly be attributed to the different ethanol metabolism rates observed between male and female animals. This is supported by our findings, which show corresponding differences in the increase in acetyl-CoA levels between males and females after ethanol administration. In males we observed a transient increase in acetyl-CoA levels as early as 15 min post-injection, while in female samples the rise in acetyl-CoA levels was detected only after 1 h of ethanol administration.

Several reports showed that αTAT1 and HDAC6 are the principal acetylase/deacetylase enzymes that modify α-tubulin. This assertion has been confirmed by studies using αTAT1 or HDAC6 null mice, respectively [27,28,29]. Interestingly, the enzyme αTAT1 exhibited a notable increase in expression within 30 min of ethanol administration in the male cerebellum. In females the higher αTAT1 levels were detected at 1 h post-injection. The rise in αTAT1 expression closely correlates with the substantial increase in acetylated α-tubulin. This observation implies that an elevation in αTAT1 expression also can play a significant role in the higher level of α-tubulin acetylation. On the other hand, there was no alteration in the expression level of the deacetylase enzyme HDAC6 in male or female mice. Our data thus suggest that the observed increase in α-tubulin acetylation was probably caused by an ethanol-induced increase in the production of acetyl-CoA and higher αTAT1 expression.

Alcohol administration upregulates histone acetyl transferase activity in the brain [30,31] and thus contributes to histone acetylation [32]. Acetylation of histone lysine residues reduces the electrostatic interaction between histone proteins and DNA, which is thought to relax chromatin structure and make DNA more accessible to transcriptional regulation [33]. Since hyperacetylation of histones in promoter regions is strongly associated with gene activation, the ethanol-induced increase in histone protein acetylation leads to changes in the expression levels of corresponding proteins [34]. This mechanism is probably responsible for our observed differences in the expression levels of αTAT1 enzyme and consequently to the modulation of α-tubulin acetylation.

This study shows changes of ethanol-induced α-tubulin acetylation only in the cerebellum. Thus, this is a limitation of this study. However, we also collected samples and data from the hippocampus. Results from hippocampal samples are part of our next study and should shed light on the possible differences in the effects of ethanol on different brain regions.

## Data Availability

The original contributions presented in this study are included in the article. Further inquiries can be directed to the corresponding author.
